# Antioxidant, Anti-inflammatory Activities and Polyphenol Profile of *Rhamnus prinoides*

**DOI:** 10.3390/ph13040055

**Published:** 2020-03-26

**Authors:** Gui-Lin Chen, Fredrick Munyao Mutie, Yong-Bing Xu, Flora Didii Saleri, Guang-Wan Hu, Ming-Quan Guo

**Affiliations:** 1 CAS Key Laboratory of Plant Germplasm Enhancement and Specialty Agriculture, Wuhan Botanical Garden, Chinese Academy of Sciences, Wuhan 430074, China; glchen@wbgcas.cn (G.-L.C.); fredrick.munyao02@gmail.com (F.M.M.); xuyongbing17@mails.ucas.ac.cn (Y.-B.X.); didiiflora@gmail.com (F.D.S.); guangwanhu@wbgcas.cn (G.-W.H.); 2 Sino-Africa Joint Research Center, Chinese Academy of Sciences, Wuhan 430074, China; 3 Innovation Academy for Drug Discovery and Development, Chinese Academy of Sciences, Shanghai 201203, China; 4 Graduate University of Chinese Academy of Sciences, Beijing 100049, China

**Keywords:** *Rhamnus prinoides*, polyphenols, antioxidant, anti-inflammatory, chemical profile

## Abstract

*Rhamnus prinoides* L’Herit (*R. prinoides*) has long been widely consumed as folk medicine in Kenya and other Africa countries. Previous studies indicated that polyphenols were abundant in genus *Rhamnus* and exhibited outstanding antioxidant and anti-inflammatory activities. However, there are very few studies on such pharmacological activities and the polyphenol profile of this plant up to now. In the present study, the antioxidant activities of the crude *R. prinoides* extracts (CRE) and the semi-purified *R. prinoides* extracts (SPRE) of polyphenol enriched fractions were evaluated to show the strong radical scavenging effects against 1,1-diphenyl-2- picrylhydrazyl radical 2,2-diphenyl-1-(2,4,6-trinitrophenyl) hydrazyl (DPPH) (0.510 ± 0.046 and 0.204 ± 0.005, mg/mL), and 2,2′-azinobis-(3-ethylbenzthiazoline-6-sulfonic acid) (ABTS) (0.596 ± 0.005 and 0.096 ± 0.004, mg/mL), respectively. Later, the SPRE with higher contents of polyphenols and flavonoids displayed obvious anti-inflammatory activities through reducing the NO production at the dosage of 11.11 − 100 μg/mL, and the COX-2 inhibitory activity with an IC_50_ value at 20.61 ± 0.13 μg/mL. Meanwhile, the HPLC-UV/ESI-MS/MS analysis of polyphenol profile of *R. prinoides* revealed that flavonoids and their glycosides were the major ingredients, and potentially responsible for its strong antioxidant and anti-inflammatory activities. For the first time, the present study comprehensively demonstrated the chemical profile of *R. prinoides*, as well as noteworthy antioxidant and anti-inflammatory activities, which confirmed that *R. prinoides* is a good natural source of polyphenols and flavonoids, and provided valuable information on this medicinal plant as folk medicine and with good potential for future healthcare practice.

## 1. Introduction

*Rhamnus prinoides* L’Herit (*R. prinoides*), belonging to family Rhamnaceae, and also commonly known as dogwood or Gesho in Amharic, is widely distributed in many countries of eastern, central, and southern Africa [1]. This plant has numerous valuable uses in local communities: the fruits are edible as a food source; the wood is hard and used as timber; and the stems and branches are usually made into ornaments, shades, fence edges, etc. [2]. Nowadays, as an important hopping agent in the beer industry, the stems and leaves of this plant are the all-important ingredients to provide the characteristic bitter flavor, and to inhibit the growth of some species of bacteria, when preparing the fermented commercial beverages, *Tella* and *Tedj*, in Ethiopia and Cameroon, and it is predicted that these prevalent beverages are consumed by over 5 million people daily in Ethiopia [1,3,4].

Although exotic to Kenya, *R. prinoides* occurs commonly in Rift-valley and Central provinces and has been used as a traditional folk herb medicine for centuries. The root decoction can be taken orally or mixed with milk as a blood purifier and gargle, and also for the treatment of the dyspepsia, flu/cold, brucellosis, rheumatism, pneumonia, stomach-ache, back pain, gonorrhea, and malnutrition [5,6]. The leaves can be made into liniment to alleviate joint sprains, and its decoction can be used for chest pain, stomach complications, fever, common cold, diarrhea, malaria, and ringworm infections [1,7]. The combination of leaves/stems can be applied for the therapy of tonsil in central Kenya [8]. Currently, an in vitro anti-plasmodium study on many plants used in Kisii, Kenya, displayed that aqueous extracts from the root bark of *R. prinoides* exerted distinct antimalarial effects against both chloroquine (CQ)-sensitive and *Plasmodium falciparum* with an IC_50_ values less than 30 µg/mL [9]. In another effort on in vivo antimalarial activities in Kenya, the hot water extracts of *R. prinoides* root barks exhibited high chemo suppression of 51% at the dosage of 500 mg/kg in mice on the CQ-resistant *Plasmodium berghei* NK65. Meanwhile, its leaf extracts showed striking parasitemia suppression and prolonged the survival of mice in a case of more than 2 weeks [10]. Besides, the sonicated aqueous extracts of *R. prinoides* root exhibited distinct acetylcholinesterase (ACE) inhibition with an IC_50_ value at 0.201 mg/mL, and showed potential utility in the treatment of Alzheimer’s disease [11]. 

Except for the essential and non-essential metals, volatile oils, and saponins with lower content, present phytochemical studies indicated that *R. prinoides* mainly contains varieties of phenolic compounds, including flavonoids, anthraquinones, naphthols, and their glycosides [1,12,13,14,15]. For example, the geshoidin, a naphthalene glycoside (β-sorigenin-8-*O*-β-D-glucoside), is one of the most important compounds in this plant, and partially responsible for the characteristic bitterness in the popular beverages of *Tella*. In addition, the discovery of this compound is of highlighted significance in studying the phytochemicals of this plant [16]. Polyphenols are the main antioxidant components in plants. On the one hand, polyphenols possess strong total reducing power, 1,1-diphenyl-2-picrylhydrazyl radical 2,2-diphenyl-1-(2,4,6-trinitrophenyl) hydrazyl / 2,2′-azinobis- (3-ethylbenzthiazoline-6-sulfonic acid) (DPPH/ABTS^+^) free radicals, and nitrite scavenging activities [17]; on the other hand, these compounds could closely be involved in many physiological processes, including but not limited to inflammation, antibiosis, hyperlipidemia, hyperglycemia, and cancer [18,19,20]; for example, polyphenols could inhibit the bioactivities of the vascular endothelial growth factor (VEGF) so as to suppress vascular regeneration [21]. As a matter of fact, however, there are still few reports about the potentials of polyphenols in *R. prinoides* in Kenya for antioxidants and their related activities. Today, more than 60% of the population in South Africa consumes traditional herbal medicines, and still up to 1.5 billion people worldwide consult the medicinal plants as their primary health care, especially in rural areas of developing countries [22]. Hence, with the growing interests in traditional herb medicines worldwide, the present study aims to comprehensively explore the antioxidant and anti-inflammatory characteristics and the polyphenol profile of the traditional ethno-medicine, *R. prinoides* in Kenya, and then further provide practical guidance for quality control (QC) and its daily and clinical usages as valuable research resource for the development of new natural pharmaceuticals. 

## 2. Results and Discussion

### 2.1. Enrichment and Phytochemical Contents

Our early work suggested that 60% ethanol solution was applicable for the extraction of polyphenols in *Rhamnus davurica* [23]. As shown in Table 1, the total phenolic content (TPC) and total flavonoids content (TFC) of the crude *R. prinoides* extracts (CRE) were 228.21 ± 13.34 gallic acid equivalents (GAE)/g and 352.25 ± 10.95 RE/g, which were eight times higher than that of dry *R. prinoides* powders at 26.86 ± 1.57 GAE/g and 41.46 ± 4.80 RE/g, respectively. Polyamide is a kind of polymer containing amide bonds (-CONH-), which can tightly absorb and bind with the compounds with hydroxyl phenols, acids, quinones, and nitro groups by the hydrogen bonds. Therefore, polyamide resin is particularly suitable for the purification and separation of the phenolic components from the complex natural products, including flavonoids, phenolic acids, quinones, carbonyl, and carboxyl compounds [24]. After purification with the polyamide resin in the present study, the TPC and TFC in semi-purified *R. prinoides* extracts (SPRE) went up to 553.67 ± 7.06 GAE/g and 958.21 ± 21.18 RE/g, showing an almost three times higher than that of in CRE. Based on the yields of CRE (11.72%) and SPRE (1.36%) to the *R. prinoides* powders, the higher TPC and TFC values in SPRE in this study also demonstrated the effective application of polyamide resin for the enrichment of the polyphenols. In this regard, the CRE (352.25 ± 10.95 mg RE/g extract) and SPRE (958.21 ± 21.18 mg RE/g extract) in this study obtained from *R. prinoides* in Kenya communities presented higher TFC values than that of the methanol extract (51.17 mg quercetin equivalent (QE)/g extract), aqueous extract (24.09 mg QE/g extract) and traditional boiling aqueous extract (12.03 mg QE/g extract) of *Rhamnus alaternus* bark from Algeria [25], and ethyl acetate (EA) extract (108.03 ± 3.09 mg catechin equivalent (CE)/g extract) of *Rhamnus lycioides* leaves from Algeria [26]. The distinct diversities of TFC values in the aforementioned extracts might be caused by the differences of extraction solvent polarities and purification processes.

### 2.2. Evaluation of Antioxidant Activities

The antioxidant activities of the CRE and SPRE were evaluated by the most frequently-used DPPH and ABTS radical scavenging tests, and all the results were expressed as IC_50_ and Trolox equivalents (TEs) and shown in Table 2. For the DPPH assays, the SPRE at IC_50_ of 0.204 ± 0.005 mg/mL exerted significantly higher scavenging activity than CRE of 0.510 ± 0.046 mg/mL (*p* < 0.01), together with the similar trend at the 2361.3 ± 57.9 μM TE/g and 945.5 ± 85.2 μM TE/g (*p* < 0.01). As for the ABTS assays, the SPRE also exhibited higher ABTS free scavenging activities compared with the CRE in regard to the IC_50_ values (0.096 ± 0.004 vs. 0.596 ± 0.005, mg/mL) and TEs (1697.9 ± 70.7 vs. 273.5 ± 2.29, μM TE/g) (*p* < 0.01). Interestingly, the SPRE displayed higher DPPH scavenging activity of IC_50_ at 0.204 ± 0.005 mg/mL and TE at 2361.3 ± 57.9 μM TE/g than the positive control butylated hydroxytoluene (BHT) of IC_50_ at 0.286 ± 0.010 mg/mL and TE at 1684.3 ± 46.3 μM TE/g, which simultaneously confirmed the antioxidant potential of *R. prinoides*.

Similar studies revealed that the bark extracts of four Rhamnus species from Croatia communities, namely *Rhamnus alaternus, Rhamnus fallax, Rhamnus intermedia,* and *Rhamnus pumila*, displayed relatively lower DPPH values of 78.7 ± 3.16, 22.3 ± 0.54, 72.2 ± 4.00, and 53.1 ± 1.57 μg/mL [27], respectively, and also the bark extracts of *Rhamnus catharticus* and *Rhamnus orbiculatus* from Dedin and Mt. Sniježnica with the efficient concentration (EC_50_) values of 64 ± 5 and 89 ± 2 μg/mL [28], respectively. Hence, the obtained antioxidant activities for the above extracts might be a result of the high TPC and TFC levels quantified in the extracts. As a matter of fact, phenolic compounds are commonly closely correlated with antioxidant activities, which might be due to their hydrogen- donating properties as free radical scavengers. Particularly, lots of well-known flavonoids, such as quercetin, kaempferol, isorhamnetin, rhamnetin, and their glycosides identified in the present study, might be attributed to those results [26].

As the active part, the H atoms in the hydroxyl groups of polyphenols can combine with the free radicals to form the polyphenol-radicals, and then react with other radicals, thus terminating the radical chain reaction [29]. In this current assay, it was assumed that the DPPH·and ABTS^+^ free radicals in the solution turned into the non-radicals of the DPPH-H and ABTS, once encountering the H atom donors of hydroxyl groups of polyphenols in *R. prinoides*. That is to say, the active H atom donors, polyphenols, in *R. prinoides* possess strong antioxidant activity by capturing the DPPH and ABTS^+^ free radicals. To this end, the SPRE was thus applied logically into the following assays.

### 2.3. Evaluation of Anti-inflammatory Activities

NO is an important inflammatory mediator secreted by activated macrophages. When produced in large quantities, NO will combine with O_2_^-^ to form peroxynitrite anion, which is an important factor leading to cell damage, energy depletion, and cell death, as well as an important link for NO to produce pathological damages [30]. COX-2 is known as a key rate-limiting enzyme that catalyzes the biosynthesis of the prostaglandins (PGs) from the precursor compound of the arachidonic acid, which induces the inflammatory cells to release chemokines and promote the movement of the inflammatory cells, thus facilitating the occurrence and metastasis of the inflammation [31]. Considering that inhibition of COX-2 activity and NO production could reduce damages to normal cells in the process of inflammation, thus significantly alleviating the typical inflammatory symptoms, they have become two of the major targets for the treatment of inflammatory diseases. Therefore, drugs that could inhibit COX-2 activity and NO production have potential anti-inflammatory activity. 

**Figure 1:**
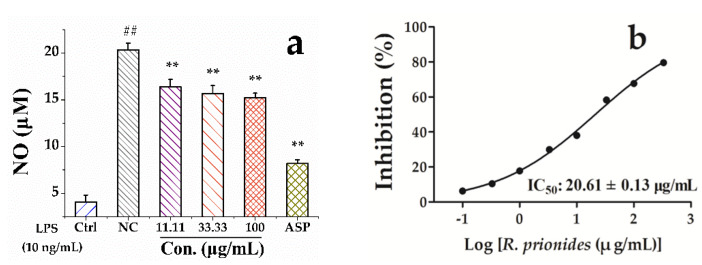
Anti-inflammatory activities of the semi-purified *R. prinoides* extracts (SPRE) of *R. prinoides* on NO production (**a**) and COX-2 inhibition (**b**). Results are expressed as the Mean ± SD (n = 3). Ctrl, normal control group; NC, negative group; ASP, aspirin; LPS, lipopolysaccharide; ** *p* < 0.01, compared with the NC group; ^##^ *p* < 0.01, compared with the Ctrl group.

**Figure 2:**
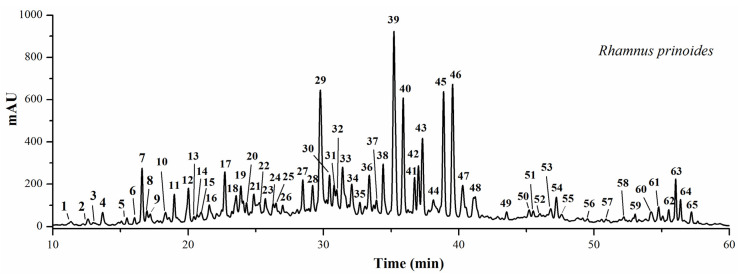
The HPLC-UV chromatogram profile of SPRE of *R. prinoides* (Kenya) at 360 nm. The peak numbers in the figure correlate to those in Table 3 below.

According to Figure 1a, compared with the negative group (NC) group, the NO production was significantly decelerated when the lipopolysaccharide (LPS)-stimulated RAW264.7 macrophage cells were incubated with the SPRE of *R. prinoides* at the concentrations of 11.11 - 100 μg/mL in a dose-dependent manner. Meanwhile, the SPRE in Figure 1b also exhibited an obvious inhibitory effect against COX-2 with an IC_50_ value of 20.61 ± 0.13 μg/mL, compared with the positive control aspirin at an IC_50_ value of 6.33 ± 0.05 μg/mL. In previous studies, the *R. davurica* leaf extract demonstrated remarkable anti-allergic activity by getting involved in the Fyn/Syk pathway in the antigen-stimulated mast cells [32]; quercetin from *Rhamnus nakaharai* and frangulin B from *Rhamnus formosana* displayed strong inhibition against the formation of TNF-α in the LPS- stimulated RAW 264.7 macrophage cells with IC_50_ values of 49.7 ± 6.1 μM and 24.2 ± 12.8 μM, respectively. Meanwhile, flavonoids of quercetin, quercetin 3-O-methyl ether, kaempferol from *R. nakaharai,* and frangulin B from *R. formosana* also displayed strong inhibition against the formation of TNF-α in the LPS/IFNγ-stimulated N9 cells with IC_50_ values of 85.6 ± 2.2 μM, 43.3 ± 10.1 μM, 11.0 ± 4.6 μM, and 42.6 ± 2.8 μM, respectively, compared with the positive control of dexamethasone at an IC_50_ value of 82.0 ± 3.8 μM [33]. In addition, 11 compounds were further screened out to be the potent COX-2 inhibitors from the bark extract of *R. davurica* with the UF-HPLC-MS hyphenated technique, in which vitexin, apigenin, and kaempferol exerted outstanding COX-2 inhibitory effect with IC_50_ values of 55.94 ± 2.59, 10.14 ± 0.45 and 9.27 ± 0.43 μg/mL, respectively [34]. However, there is no such report about the anti-inflammatory applications in floks of *R. prinoides* in Kenya until now. Evidences from the HPLC-MS analysis in Figure 2 and Table 3 revealed that the above-mentioned flavonoids and their glycosides were also contained in the *R. prinoides* extracts from Kenya communities. For the first time, hence, the present results proved the noteworthy anti-inflammatory effects of *R. prinoides* by inhibiting the COX-2 activity and NO production in RAW264.7 cells. 

### 2.4. HPLC-UV/ESI-MS/MS Analysis

A chemical fingerprint profile can comprehensively reflect the types and quantities of chemical components contained in medicinal plants and their products, and then describe and evaluate their quality as a whole. Therefore, it can be used to analyze the authenticity, goodness, and stability of the quality of medicinal plants and their products [23]. By combining the high separation performance of HPLC to complex samples, with high selectivity, sensitivity, and the ability to provide molecular weight and structure information of MS, the hyphenated HPLC-MS is very suitable for the comprehensive evaluation of the QC of medicinal plants [35,36]. *R. prinoides* has been widely used in Kenyan local communities to treat a variety of diseases. However, studies on the chemical compositions of this plant and its products remain rare, and the lack of research in this field cannot guarantee the clinical efficacy of this plant and its products. Therefore, current studies are urgently needed to analyze the chemical constituents of this medicinal plant comprehensively. 

In present study, the HPLC-UV chromatogram profile of *R. prinoides* (Kenya) was implemented by using the HPLC-UV/ESI-MS/MS. As shown in Figure 2 above, 65 peaks were detected in SPRE. Further structural identification and characterization of those compounds in Table 3 were carried out strictly by comparison with the chemical standards or the MS fragments reported in previous studies [12,13,14,15,23,37,38]. As a result, several types of polyphenols, including flavonoids and their glycosides (peaks 3–9, 11–15, 18, 19, 21, 22, 24–26, 28–31, 34, 37, 38, 40–42, 44–50, 53, 54, 56–63, 65), phenols and their glycosides (peaks 1, 2, 20, 23), naphthols and their glycosides (peaks 16, 36, 39, 43, 51, 52), anthraquinones and their glycosides (peaks 17, 33, 35, 64), phenylpropanoid glycoside (peak 55), and saponin (peak 32), are revealed majorly in *R. prinoides*. On this basis, further studies on spectrum-effect should also be carried out to truly correlate its QC with its clinical efficacy, and help to clarify the mechanisms of action. For the relative quantitative analysis of chemicals in SPRE, the aforementioned flavonoids, phenols, naphthols, anthraquinones, phenylpropanoid, saponin, and their glycosides take up 69.54%, 2.39%, 17.87%, 6.39%, 0.53%, 0.98%, respectively. Most interesting, geshoidin, a naphthalenic glucoside, holds the highest content, which might be the reason that it acts as the basic bittering ingredient for the popular beverages of *Tella* and *Tedj* in Ethiopia and Cameroon [3]. Besides, geshoidin also exhibited outstanding superoxide anion (O_2_^●-^) scavenging activity in vitro with an IC_50_ value at 1.9 mM, compared with the ascorbic acid (Vc) at 1.7 mM [16]. 

Therefore, for the polyphenol profile of *R. prinoides*, in this regard, both the flavonoids profiling of the main components and the highest ingredient of geshoidin (iconic marker) strongly provide the scientific basis for the quality assurance and functional components of this medicinal and edible plant. Coincidentally, our earlier study also indicated that antioxidant activities correlated closely with the higher content of flavonoids in *R. davurica* [23]. In other words, the higher TFC values in SPRE further demonstrated the potent stronger antioxidant and anti-inflammatory activities of *R. prinoides* in the present study. 

## 3. Materials and Methods 

### 3.1. Chemicals and Reagents

The chemical standards of rutin, gallic acid, penicillin, streptomycin, aspirin (ASP), and lipopolysaccharide (LPS) were bought from Aladdin Industrial Corporation (Shanghai, China). Butylated hydroxytoluene (BHT), 6-hydroxy-2,5,7,8-tetramethyl-chroman-2-carboxylic acid (Trolox), 1,1-diphenyl-2-picrylhydrazyl radical 2,2-diphenyl-1-(2,4,6-trinitrophenyl) hydrazyl (DPPH), 2,2’- azinobis-(3-ethylbenzthiazoline-6-sulfonic acid) (ABTS) were provided by Sigma–Aldrich Corp. (St. Louis, USA). MTT (3-(4,5-dimethyl-2-thiazolyl)-2,5-diphenyl-2-H-tetrazolium bromide), FBS (fetal bovine serum), and DMEM (Dulbecco’s Modified Eagle Medium) were purchased from Gibco (Life Technologies, USA). Ultra-pure water was prepared with the EPED system (Nanjing Yeap Esselte Technology Development Co., Nanjing, China). All the other analytical grade chemicals and solvents were purchased from Shanghai Chemical Reagent Corp. (Shanghai, China).

### 3.2. Plant Material and Sample Preparation

The fresh stems and stem barks of *R. prinoides* (3.0 kg) were collected randomly from a 10,000 m^2^ area of Mount Kenya (Kenya) in July of 2018. Afterward, the specimens of those plant materials were kindly authenticated by Professor Guangwan Hu, a senior taxonomist from the Key Laboratory of Plant Germplasm Enhancement and Specialty Agriculture (Wuhan Botanical Garden, Chinese Academy of Sciences). A voucher specimen was stored in the herbarium of the Key Laboratory.

For the sample preparation, the air-dried plant materials were firstly ground to a powder with a high-speed disintegrator. Then, an aliquot of 100 g accurately weighted plant powders was extracted ultrasonically three times using 60% ethanol at room temperature for 30 min to produce the crude *R. prinoides* extracts (CRE). Then the CRE were dispersed in water and extracted with petroleum ether (PE) and EA, successively. Finally, the EA extracts were loaded into a polyamide column, and the targeted semipurified *R. prinoides* extracts (SPRE) of the polyphenols enriched fraction was prepared by eluting with 80% ethanol solution. The yields of CRE and SPRE were referred to the percentage of the dry weight of the extracts to the dry plant samples, and calculated based on the formula below:

(1) $$\mathrm{Yield}\;(\%)=\frac{\mathrm{Weight}\;\mathrm{of}\;\mathrm{dry}\;\mathrm{extract}}{\mathrm{Weight}\;\mathrm{of}\;\mathrm{dry}\;\mathrm{plant}\;\mathrm{samples}}\times 100$$

### 3.3. Determinations of Phenolic Constituents 

#### 3.3.1. Determination of Total Phenolic Content (TPC)

The total phenolic contents in the above two extracts were measured using the Folin–Ciocalteu method [39] with some modifications. In brief, 200 µL of the properly diluted sample solutions or the standard gallic acid (GA) solutions were firstly mixed with an equivalent Folin–Ciocalteu reagent (0.25 M) by vortexing. After that, 1000 µL of Na_2_CO_3_ (1.0 M) and 600 µL of H_2_O were added and mixed gently. Later, the reaction mixtures were further cultivated for 1 h at room temperature in the dark, and finally, the absorbed optical density (AOD) was recorded at the wavelength of 760 nm. The GA was served as the standard, and the TPC was defined as milligram of GA equivalents per gram of the sample (mg GAE/g).

#### 3.3.2. Determination of Total Flavonoid Content (TFC)

The total flavonoid content in all samples was determined with the previous colorimetric report [40] using rutin as a standard flavonoid compound. In brief, 180 µL of the properly diluted extracts or rutin standard solutions were firstly mixed with 1080 µL of H_2_O and subsequently with 60 µL of 5% NaNO_2_ solution. After incubation for 6 min, 120 µL of 10% AlCl_3_ solution was then added and left to stand for 6 min. After that, 360 µL of 4% NaOH solution was added, and these reaction mixtures were kept for another 15 min at room temperature. The AODs of these reaction mixtures were detected at the wavelength of 510 nm, and the TFC was defined as milligram of rutin equivalents per gram of the sample (mg RE/g).

### 3.4. Determinations of Antioxidant Activity

#### 3.4.1. DPPH Free Radical Scavenging Activity

The DPPH free radical scavenging assay was carried out in 96-well microliter plates according to a previously described method [41]. Briefly, 10 μL of the Trolox or sample solutions at various concentrations were mixed with 190 μL of the DPPH methanol solution (100 μM, final concentration) in the 96- well plate. Then, the reaction mixtures were shaken gently and cultivated in the dark for 0.5 h at room temperature. Thereafter, the discoloration of DPPH radicals was detected through recording the AOD at the wavelength of 517 nm with a Tecan microplate reader (Infinite M1000, Switzerland). BHT was used as the positive control. The DPPH radical scavenging activity (RSA) was calculated as the equation: RSA (%) = [(Acontrol − Asample)/Acontrol] × 100%. Each sample solution test was repeated three times, and all the results of the two flavonoid fractions were described to be micromolar Trolox equivalents (TE) per gram of the sample (μM TE/g).

#### 3.4.2. ABTS Free Radical Scavenging Activity

The ABTS radical scavenging activity was assessed using a previous method [41]. In brief, the ABTS^+^ stock solution was first prepared by mixing the ABTS solution (7.0 mM, in H_2_O) and an equal volume of the potassium persulfate solution (2.45 mM, in H_2_O) in darkness for 12–16 h. After that, the ABTS^+^ working solution was prepared through successively diluting the ABTS^+^ stock solution with 80% ethanol to the AOD of 0.70 ± 0.01 at the wavelength of 734 nm. The reaction mixture was composed of 10 μL of the Trolox or sample solution and 190 μL of the ABTS^+^ working solution, and then cultivated in the dark for 0.5 h. The AOD at the wavelength of 734 nm was obtained in triplicate. The calculation and expression of ABTS were consistent with the above mentioned DPPH radical scavenging activity.

### 3.5. Determination of Anti-inflammatory Activities

#### 3.5.1. Measurement of LPS-stimulated NO in Macrophage RAW 264.7 Cells

The RAW264.7 macrophage cells were purchased from the American Type Culture Collection (ATCC). The cells were maintained at 37 °C in DMEM medium supplemented with the 10% FBS, 1% penicillin–streptomycin with 5% CO_2_ in a humidified incubator. The nitrite concentration, an indicator of the NO synthesis, was measured based on the previously described Griess reaction method [42]. Briefly, RAW 264.7 cells were transferred into the 96-well plate with a density of 5 × 10^4^ cells/well and kept for 24 h. These cells were incubated with various concentrations of the SPRE solution for 2 h and then followed by the incubation with 10 ng/mL of LPS for another 24 h. Meanwhile, 10.0 μg/mL of aspirin was used as the positive control. After that, 100 μL of the culture supernatant was cultivated with the same volume of Griess reagent for 15 min at room temperature, and the AOD was finally determined with a microplate reader at the wavelength of 540 nm, according to the manufacturer’s instructions. 

#### 3.5.2. COX-2 Inhibition Assay

Along with the aspirin used as the positive control, the in vitro COX-2 inhibitory test was implemented based on our previous method [34]. Briefly, COX-2 (1U, 20 μL) was mixed with Tris-HCl (100 mM, 150 μL) and hematin (1.0 μM, 10 μL) and shaken gently for 2.0 min. After that, 10 μL of the tested sample solutions (0.46 − 333 μg/mL) was added and incubated for 5.0 min. Then, arachidonic acid (100 μM, 10 μL) and TMPD (10 μM, 10 μL) were added to the initiated the reaction, and finally, the reaction mixtures were terminated by the addition of HCl solution (2.0 M, 20 μL) after incubation for 5.0 min. The optical values (OD) of the reaction mixtures were monitored at the wavelength of 590 nm. The IC_50_ value and its dose-dependent curve of the SPRE sample were acquired based on the non-linear regression analysis (GraphPad, v5.01). The data were expressed as Mean ± SD of three replicates.

### 3.6. HPLC-UV/ESI-MS/MS Analysis of R. prinoides 

The HPLC-UV/ESI-MS/MS Analysis of *R. prinoides* was implemented by using the Termo Accela 600 HPLC system, which was connected with the TSQ Quantum Access MAX mass spectrometer (Termo Fisher Scientific, San Jose, CA, USA). A Waters SunFire™ RP-C18 column (150 mm × 4.6 mm, 3.5 µm) was employed for the chromatographic separation. The H_2_O (A) and acetonitrile (B) were used as the mobile phases, and the elution gradient was set as follows: 0–2 min, 15% B; 2–45 min, 14% − 45% B; 45–55 min, 45% − 60% B; 55–60 min, 60% − 15% B. The injection volume was 10 μL, the flow rate was 0.5 mL/min, the column temperature was kept at 30 °C, and the on-line UV chromatograms were monitored at 360 nm. The relative quantitative analysis was calculated by the proportion of the AUC of one component in the HPLC-UV chromatogram profile to the total AUC of all components. For the following ESI-MS/MS analysis, the MS/MS conditions were set the same as those of our previous study [23].

### 3.7. Statistical Analysis

All the statistical analysis was performed using the SPSS program (V 16.0). One-way ANOVA with the Tukey and LSD tests was employed to compare the significance between groups. All the values are present to be the Mean ± SD (standard deviation, n = 3), and the significant differences were considered at *p* ≤ 0.05 and *p* ≤ 0.01.

## 4. Conclusions

In the present study, the noteworthy antioxidant and anti-inflammatory activities of the polyphenols of *R. prinoides* from Mount Kenya were comprehensively evaluated for the first time. As a result, the polyphenol enriched extracts of CRE and SPRE displayed strong antioxidant activities by capturing the DPPH and ABTS^+^ free radicals. Along with the higher contents of polyphenols and flavonoids, and the stronger antioxidant activities, the SPRE showed potential anti-inflammatory activities by reducing the NO production and the COX-2 activity. On the other hand, further HPLC-UV/ESI-MS/MS analysis of the polyphenol profile of *R. prinoides* revealed that flavonoids and their glycosides not only made up the major ingredients but were also potentially responsible for its strong antioxidant and anti-inflammatory activities. In conclusion, the present study comprehensively demonstrated that the high content of polyphenols in *R. prinoides*, along with noteworthy antioxidant and anti-inflammatory activities, confirmed that *R. prinoides* is a good natural source of polyphenols and flavonoids, and provided further scientific evidences for its use as folk medicine and with good potential for future healthcare practice.

## Figures and Tables

**Table 1:** Total phenolic content (TPC) and total flavonoids content (TFC) of the extracts and fractions of *R. prinoides*.

	TPC (mg GAE/g)	TFC (mg RE/g)
CRE	26.86 ± 1.57 ^a^	41.46 ± 4.80 ^a^
	228.21 ± 13.34 ^b^	352.25 ± 10.95 ^b^
SPRE	553.67 ± 7.06 ^c^	958.21 ± 21.18 ^c^

Results are expressed as the Mean ± SD (n = 3). CRE, crude *R. prinoides* extracts; SPRE, semipurified *R. prinoides* extracts; TPC, total phenolic content; TFC, total flavonoids content; GAE, gallic acid equivalents; RE, rutin equivalents. ^a^ Based on *R. prinoides* powders. ^b^ Based on CRE. ^c^ Based on SPRE.

**Table 2:** IC_50_ Values of the crude *R. prinoides* extracts (CRE) and the semi-purified *R. prinoides* extracts (SPRE) of *R. prinoides* on the 2,2-diphenyl-1-picrylhydrazyl (DPPH) and 2,2′-Azinobis- (3-ethylbenzthiazoline-6-sulfonic acid) (ABTS) free radicals.

Sample	DPPH		ABTS	
	IC_50_ (mg/mL)	μM TE/g	IC_50_ (mg/mL)	μM TE/g
CRE	0.510 ± 0.046	945.5 ± 85.2	0.596 ± 0.005	273.5 ± 2.29
SPRE	0.204 ± 0.005 **	2361.3 ± 57.9	0.096 ± 0.004 **	1697.9 ± 70.7
BHT	0.286 ± 0.010	1684.3 ± 46.3	0.059 ± 0.003	2762.7 ± 71.2
Trolox	0.121 ± 0.005	ND	0.041 ± 0.002	ND

Results are expressed as the Mean ± SD (n = 3). DPPH: 2,2-diphenyl-1-picrylhydrazyl; ABTS: 2,2′-Azinobis-(3-ethylbenzthiazoline-6-sulfonic acid); BHT, butylated hydroxytoluene; CRE, crude *R. prinoides* extracts; SPRE, semipurified *R. prinoides* extracts; half maximal inhibitory concentration (IC_50_) value represents the concentration that leads to 50% decrease in the scavenging of DPPH or ABTS radical; μM TE/g, μM Trolox equivalents per gram of sample; ND, Not detected. ** *p* < 0.01, compared with CRE.

**Table 3:** The HPLC-ESI-MS/MS data of the detected polyphenol profile of SPRE of *R. prinoides* (Kenya).

Peak No.	Rt (min)	[M–H]^−^ (m/z)	MS/MS ions	Tentative Identification	AUC (%)
1	11.33	341	341, 221, 179, 131, 119	Caffeic acid 4-*O*-hexoside ^a^	0.31
2	12.60	153	153, 109, 108	Protocatechuic acid ^a^	0.33
3	12.98	447	447, 285, 229, 207, 165, 137	Kaempferol 3-*O*-glucoside ^a^	0.18
4	13.68	305	305, 221, 179, 165, 125	Gallocatechin ^a^	0.68
5	15.48	577	577, 289, 245, 161, 125	Proanthocyanidin B3 ^a^	0.31
6	16.04	447	447, 327, 285, 284, 255, 227	Luteolin 5-*O*-glucoside ^a^	0.35
7	16.60	769	769, 623, 315, 299	Isorhamnetin 3-*O*-rhamninoside ^a^	2.03
8	16.95	483	483, 356, 353, 337, 239, 195, 127	Quercetin 3-*O*-methyl ether peracetate ^a^	0.48
9	17.20	303	303, 285, 241, 217, 151, 125	Taxifolin ^b^	0.42
10	18.33	597	597, 465, 422, 353, 241, 209	Unknown	0.61
11	18.98	447	447, 284, 284, 255, 227	Kaempferol 7-*O*-glucoside ^a^	1.09
12	20.02	287	287, 259, 215, 151, 125	Aromadendrin ^a^	1.86
13	20.43	811	811, 315, 300, 271	Rhamnetin 3-*O*-acetyl-rhamninoside ^a^	0.28
14	20.67	431	431, 341, 311, 283, 269	Apigenin 8-*C*-glucoside (Vitexin) ^b^	0.33
15	20.96	463	463, 301, 300, 243, 179	Quercetin-3-*O*-glucoside (Isoquercetin) ^b^	0.71
16	21.57	377	377, 257, 215, 187	β-Sorigenin 1-*O*-glucoside ^a^	1.14
17	22.72	445	445, 283, 268, 239	Physcion 8-*O*-glucoside ^a^	1.99
18	23.55	463	463, 301, 300, 271, 255, 179	Quercetin 7-*O*-glucoside ^a^	1.18
19	23.90	465	465, 303, 273, 223, 181	Dihydroquercetin 3-O-glucoside ^a^	1.44
20	24.31	181	181, 137, 93	4-(2-Hydroxyethoxy) benzoic acid ^a^	0.75
21	24.85	431	431, 269, 265, 241	Apigenin 7-*O*-glucoside ^a^	1.15
22	25.26	449	449, 359, 286, 257, 227, 171	Maesopsin 6-*O*-glucoside ^a^	0.84
23	25.70	195	195, 151, 107, 91	4-(2-Hhydroxyethoxymethyl) benzoic acid ^a^	1.00
24	26.28	477	477, 315, 299, 254, 243	Quercetin 3-methyl ether 7-O-galactoside ^a^	0.63
25	26.49	781	781, 635, 285, 255	Rhamnocitrin 3-*O*-rhamninoside ^a^	0.63
26	26.98	473	473, 327, 307, 165, 145	Kaempferol 3,5,7-trimethyl ether 4’-*O*-pentoside ^a^	0.67
27	28.48	1017	1017, 677, 451, 225, 207	Unknown	1.69
28	29.20	503	503, 327, 323, 193, 179, 165	Kaempferol 3,5,7-trimethyl ether 4’-*O*-glucuronide ^a^	1.59
29	29.77	287	287, 269, 259, 243, 151, 125.	Eriodictyo ^a^	7.62
30	30.45	595	481, 301, 287, 257, 179, 125	Aromadendrin 3-rutinoside ^a^	1.64
31	30.79	595	595, 567, 301, 287, 283, 259	Quercetin diglycoside ^a^	1.05
32	30.98	853	853, 751, 623, 315, 299, 271	Hovenidulcioside B1	0.98
33	31.42	415	415, 253, 215, 209	Chrysophanol 8-*O*-glucoside ^a^	2.95
34	32.09	461	461, 315, 213, 151	Rhamnetin 3-*O*-rhamnoside ^a^	1.73
35	32.69	563	563, 417, 311, 269, 225	Emodin 8-*O*-glucophyranosyl (6-1)-*O*-xylopyranoside ^a^	0.94
36	33.39	215	215, 187, 171, 159	β-sorigenin ^a^	2.31
37	33.92	251	251, 219, 207, 179, 135	4’-Methoxyflavone ^a^	0.80
38	34.41	609	609, 463, 301, 205, 183	Rutin ^b^	2.58
39	35.21	377	376, 332, 215, 201, 173	Geshoidin ^a^	9.62
40	35.90	433	433, 313, 283, 271, 201	Naringenin 8-*C*-glucoside ^a^	5.17
41	36.74	825	825, 783, 329, 313, 285	Rhamnazin 3-*O*-acetyl-rhamninoside ^a^	1.59
42	37.02	271	271, 177, 151, 119	Naringenin ^a^	2.07
43	37.32	539	539, 377, 257, 215, 187, 161	α-Sorinin ^a^	3.55
44	38.13	865	865, 781, 575, 327, 285	Procyanidin trimer ^a^	1.39
45	38.88	301	301, 283, 245, 151, 125	Quercetin ^b^	5.79
46	39.55	431	431, 311, 281, 265	Apigenin 6-*C*-glucoside (Isovitexin) ^b^	6.45
47	40.30	461	461, 299, 283, 265	Diosmetin 7-*O*-glucoside ^a^	2.74
48	41.22	301	301, 283, 245, 179, 151, 125	Herbacetin (8-Hydroxykaempferol) ^a^	2.31
49	43.53	593	561, 298, 285, 241, 225	Kaempferol 3-*O*-robinoside ^a^	0.77
50	45.20	757	757, 595, 372, 193, 179	Quercetin triglycoside ^a^	0.76
51	45.50	523	523, 377, 215, 187, 171	β-Sorigenin-8-rutinoside ^a^	0.68
52	45.99	553	553, 469, 283, 259, 243, 179	6-Methoxy-sorigenin-8-rutinoside ^a^	0.57
53	46.79	473	473, 311, 293, 269, 225	Sideroxylin 4′-*O*-glucoside ^a^	0.98
54	47.21	473	473, 269, 253, 241, 225	Sideroxylin 5-*O*-glucoside ^a^	1.27
55	47.59	581	581, 357, 193, 179, 161, 135	Lyoniresinol 3-*O*-glucopyranoside ^a^	0.53
56	49.51	317	287, 273, 227, 193, 105	Myricetin ^a^	0.36
57	50.88	285	285, 257, 241, 223, 197	Luteolin ^b^	0.29
58	52.20	269	269, 241, 225, 181, 169	Apigenin ^b^	0.41
59	53.03	285	285, 257, 241, 223, 197	Kaempferol ^b^	0.52
60	54.21	285	285, 241, 225, 211, 165	Sakuranetin ^a^	0.77
61	54.78	315	315, 300, 271, 171, 151	Rhamnetin ^b^	0.73
62	55.52	299	299, 284, 271, 165	Rhamnocitrin ^a^	0.64
63	56.04	329	329, 313, 285, 227, 209, 167	Rhamnazin ^a^	1.41
64	56.40	283	283, 268, 239, 211, 117	Physcion ^b^	0.81
65	57.20	315	315, 300, 287, 193, 165, 121	Rhamnetin isomer ^a^	0.53

^a^ Identification and characterization by comparison with chemical standards; ^b^ Identification and characterization by comparison with the MS fragments reported in previous studies. Rt, retention time; AUC, area under the curve.
